# Comparison of the Fracture Resistance of Root-Canal-Treated Premolars Obturated With Dissimilar Materials: An In Vitro Study

**DOI:** 10.7759/cureus.49426

**Published:** 2023-11-26

**Authors:** Mohamed Samir A Elnawawy, Fawaz Pullishery, Mustafa Hussein Alattas, Tawassul A Gerfa, Batool O Khambashi, Haneen T Almahallawi, Ghaidaa N Baghdadi, Tamer D Abdelaziz

**Affiliations:** 1 Operative Dentistry and Endodontics, Dentistry Program, Batterjee Medical College, Jeddah, SAU; 2 Dental Public Health, Dentistry Program, Batterjee Medical College, Jeddah, SAU; 3 Conservative Dental Science and Endodontics, Qassim University, Buraydah, SAU; 4 Dentistry, Dentistry Program, Batterjee Medical College, Jeddah, SAU; 5 Chemistry, Preparatory Year Program, Batterjee Medical College, Jeddah, SAU

**Keywords:** endodontic treatment outcomes, patient experience, fracture resistance, endodontic sealers, clinical durability

## Abstract

Introduction

The utilization of endodontic sealers is of paramount importance in ensuring the sustained efficacy and resilience of endodontic treatment. The primary objective of the research was to appraise and contrast the fracture resistance (FR) of three distinct categories of endodontic sealing materials that are frequently employed in the context of endodontic therapy.

Materials and methods

This research used an in vitro experimental design. Sixty single-rooted human teeth indicated for extraction were utilized according to established protocols. These teeth were then arbitrarily divided into four piles, one for each of the four possible sealants. All specimens were put through a standardized thermal cycling procedure to simulate clinical conditions after the root canals were obturated. The subsequent step involved testing the FR of each group by utilizing a universal testing machine up until failure. Any statistically significant difference in FR among the three sealing materials was identified through appropriate statistical analysis.

Results

Group 1, which utilized a particular sealing material, exhibited the highest mean fracture resistance, measuring at an impressive 1198.33 ± 321.4 Newtons (N). A post hoc analysis was done to see the exact differences between each group and statistically significant differences between Groups 1 and 2 (p<0.05), Groups 1 and 4 (p<0.05), and Groups 3 and 4 (p<0.001) were observed.

Conclusion

The FR of Group 1 specimens were noticeably greater than those of Group 2 and Group 4 while the FR of Group 3 specimens was more than that of the Group 4 specimens. This study provides important insights into the fracture resistance of various endodontic sealing materials.

## Introduction

The restoration of root canal-treated premolar teeth is a critical aspect of modern dentistry. Root canal treatment aims to eliminate infection and preserve the tooth, but it often leaves the tooth structurally compromised. In such cases, obturation, the process of filling and sealing the root canal, becomes essential to maintain the tooth's functionality and prevent potential fractures [1]. The choice of obturation materials can significantly impact the fracture resistance (FR) of the tooth. The primary goal of this procedure is to remove germs from the canals and build a three-dimensional fluid-impermeable obturation that extends from the coronal intra-orifice to the apical constriction [2]. Adhesive restorations that cover the cusps directly or indirectly can significantly enhance the FR of teeth that have undergone endodontic treatment. Endodontic obturation materials bond to the radicular dentin and may help improve the FR of the obturating materials [3]. When instrumented but not obturated canals are compared to sealers and lateral condensation, the roots are significantly strengthened [4]. Resilon and Gutta Percha root canal fillings, which have a lower modulus of elasticity than dentine, have little or no root-strengthening capacity [5]. For these reasons, the primary goal of endodontic treatment should be to strengthen any remaining tooth structure rather than to remove tooth decay. Bonded composite restorations are ideal for coronal restorations on endodontically treated teeth because they increase fracture resistance [6].

According to Roghanizad and Jones, the root canal orifice should have a three-millimeter layer of gutta-percha removed and replaced with a restorative substance [7]. The use of intracoronal barriers to prevent coronal micro-leakage has been promoted. Restorative materials with elastic moduli comparable to dentin may also provide stiffness against root fractures [3]. The comparative approach of analyzing multiple obturation materials provides valuable insights into their respective strengths and weaknesses. Practitioners can benefit from the information presented, as it aids in making informed decisions when selecting the most suitable material for their patients. Additionally, the study's focus on fracture resistance addresses a crucial concern in root canal-treated premolars, as fractures can lead to the loss of the tooth and subsequent complications. The endodontic therapeutic procedures attempt to strengthen the root dentin. As a result, using a root canal sealer while also fortifying the root against fracture makes sense. Due to the growing interest in strengthening the root canal system, adhesive root canal sealers that may enhance fracture resistance have been developed. The material's adherence and mechanical interlocking with the root dentin are anticipated to strengthen the residual tooth structure and minimize fractures. Gutta-percha (GP) has been deemed to be the preferred obturating material. The epoxy resin-based sealants AH+ and GP can be utilized interchangeably [8]. Recently, Resilon-Epiphany, a resin-based obturation substance, has been evolved. For many years, gutta-percha (GP) was the preferred root canal filler. AH+ is an epoxy resin-based sealant that is widely used in conjunction with GP. The epoxy resin-based sealants AH+ and GP can be used interchangeably. To make a sturdy monoblock, a thermoplastic synthetic resin substance that can cure in two different temperatures and a self-etching primer are mixed together. Thus, the study aimed to appraise the FR of three different endodontic sealants.

## Materials and methods

**Figure 1:**
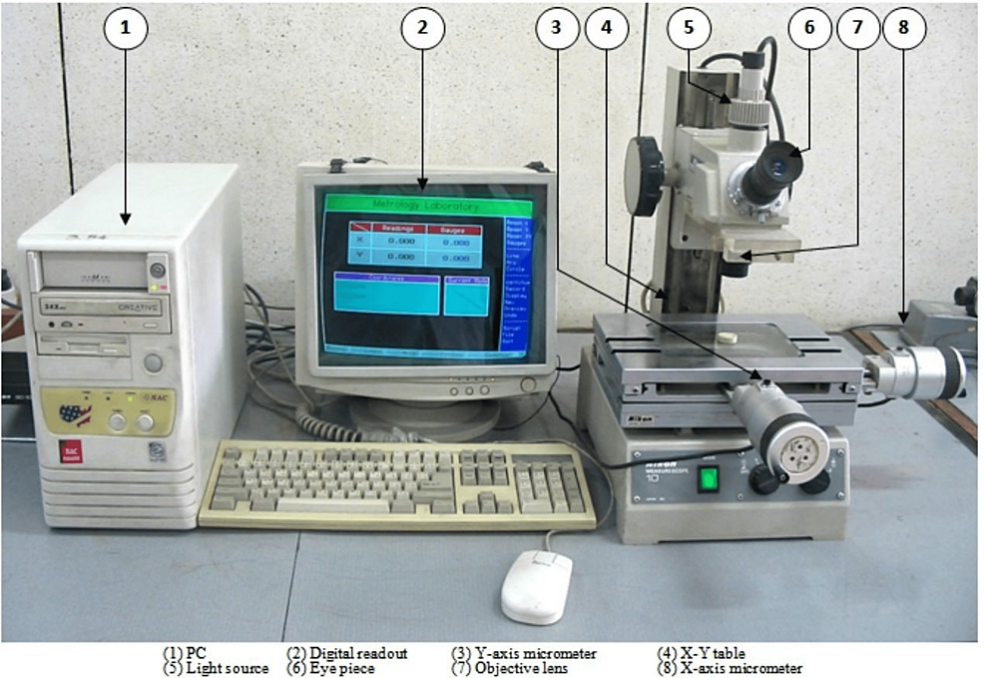
Stereomicroscope-10 Nikon Nikon Instruments Inc., Melville, NY, USA

The study was granted ethical clearance from the institutional review board (IRB) of Batterjee Medical College, Jeddah (RES-2022-0068). Sixty healthy human mandibular premolars that were slated for orthodontic extraction were the subject of the investigation. First, soft debris was removed from the tooth surface with an ultrasonic scaling instrument. The teeth were next observed at a magnification of 25X using a stereo microscope (Figure 1) to exclude those teeth with preexisting radicular fractures, craze lines, or fissures. The chosen teeth were kept in normal saline at ambient temperature to prevent them from drying out. To standardize the teeth, a 13 mm measurement was used. An endodontic access canal file of size 10 K was employed to assess apical foramen patency. The apical foramen evaluation was performed using endodontic access canal files of size 10 K, with the predefined working canal length of 1 mm below the apical foramen. The canals were instrumented to size F3 using the rotating ProTaper device, corresponding to an apical diameter of 30. Between filings, a 5.25% sodium hypochlorite irrigant was applied. The canals were irrigated with 17% EDTA (ethylenediaminetetraacetic acid) and 10% sodium hypochlorite solution for three minutes to remove the smear layer. The sterile water was used for the final rinse after waiting for five minutes and then dried using the paper points. The specimens are then assigned to one of four groups of 15 teeth randomly. Each group represents a different obturating material.

**Figure 2:**
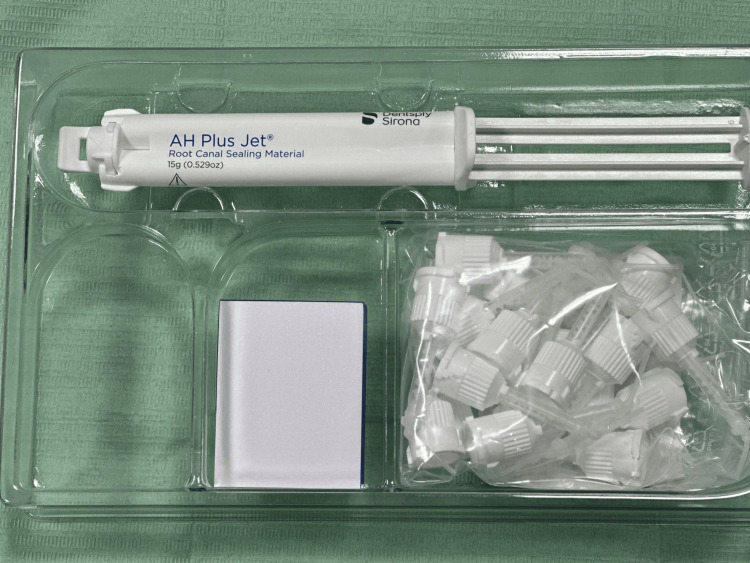
AH+ sealer

Group 1: The lateral condensation technique was employed to obturate the teeth after mixing AH+ and gutta-percha (Figure 2).

Group 2: Obturation with the lateral condensation approach with the Resilon-Epiphany System.

Group 3: Obturation using the lateral condensation approach and sealed with AH 26.

Group 4: This group of teeth remained without any obturation.

To assess fracture strength, a self-curing acrylic resin block was prepared, revealing 8 mm of the roots' length when the roots were installed vertically. The fracture strength was tested using a universal machine (Hounsfield test equipment, Model H5K5; Analytical Laboratory Services, UK). Each tooth's fracture resistance was measured in Newton.

The data of each group were summarized using descriptive statistics. The Shapiro-Wilk test was used to check the normality of the sample. Measures such as mean and standard deviation were calculated to understand the central tendency and variability of the fracture resistance values. The means of the four groups of sealers were contrasted using the one-way analysis of variance (ANOVA) followed by Tukey's HSD test for multiple comparisons. A p-value below the predetermined significance level of 0.05 was deemed to be statistically significant.

## Results

The differences in FR between the four sealant groups are shown in Table 1.

**Table 1:** Comparison of fracture resistance between four groups * a p value <0.05 is considered statistically significant.

Sealer Type	Sample size	Fracture resistance	P value
Group 1 (AH plus)	15	1198.33 ± 321.4	0.001*
Group 2 (Resilon-Epiphany)	15	873.70 ± 276.6	
Group 3 (AH 26)	15	987.01± 381.1	
Group 4 (No sealer)	15	724.52 ± 241.2	

The mean FR was found to be higher in AH plus (1198.33 ± 321.4 N) and the least was found in no sealer Group 4 (724.52 ± 241.2 N), which showed a statistically significant difference (p<0.001). A post hoc analysis was done to see the exact differences between each group (Table 2).

**Table 2:** Post hoc analysis

Groups	Differences	95% Confidence interval	P value
Group 1 vs Group 2	-324.63	-623.90 to -25.36	0.028
Group 1 vs Group 3	-211.32	-510.59 to 87.95	0.252
Group 1 vs Group 4	-473.81	-773.08 to -174.54	<0.001
Group 2 vs Group 3	113.31	-185.96 to 412.58	0.748
Group 2 vs Group 4	-149.18	-448.45 to 150.09	0.554
Group 3 vs Group 4	-573.8100	-863.90 to -283.71	0.105

The analysis showed a statistically significant difference between Groups 1 and 2 (p<0.05), Groups 1 and 4 (p<0.05), and Groups 3 and 4 (p<0.001). However, there were no statistically significant differences observed between Groups 1 and 3, Groups 2 and 3, Groups 2 and 4, and Groups 3 and 4 (p>0.05).

## Discussion

The mechanical interlocking and bonding of root canal sealants are believed to foster strength in the remaining tooth structure and increase the FR. Therefore, several research approaches have generated materials that enable adhesion to the root canals [9]. Based on the study findings results, Group 3 (AH+ sealer) had the highest fracture resistance of all the sealers tested. They may have better fracture resistance because they can enter the dentinal tubule. These characteristics are crucial for root canal sealants [10]. Additionally, the epoxy resin-based AH Plus sealant has the added benefits of easy handling and perhaps increasing the wettability of the dentin and GP surfaces. Our study findings agree with the findings of Dibaji et al., which demonstrated improved fracture resistance for AH+ and then bioceramic-based endodontic sealers such as TotalFill BC sealer [11]. Some other studies have shown superior fracture resistance for bioceramic-based endodontic sealers compared to AH+ sealers [4,12]. Additionally, Topcuolu et al. showed a higher bioceramic FR, despite the fact that the difference between the groups failed to achieve statistical significance [13]. This disparity may be attributable to the various approaches taken in the two studies.

The AH+ sealer has excellent flow characteristics, which allow it to penetrate and seal even the finest and most intricate anatomical irregularities within the root canal system. Having such a strong barrier between the root canal and the external environment helps reduce the likelihood of reinfection and root canal failure [14]. The AH+ sealer's exceptional adhesion to a wide variety of surfaces is due to its epoxy resin composition. A resilient bond is established between the root canal walls and the gutta-percha points employed for the purpose of filling the root canal space. This adhesive bond enhances the overall stability and resistance of the root filling [15]. The AH+ sealer maintains its dimensional stability over time, meaning it does not significantly shrink or expand during the setting process. This characteristic helps minimize gaps between the root canal walls and the sealants, ensuring a more uniform and consistent filling that can withstand occlusal forces better [16]. The AH+ sealer is radiopaque, which means it appears clearly on dental X-rays. This feature allows for easy post-treatment evaluation and identification of any voids or gaps in the root filling to be corrected if necessary [17]. The sealer's chemical resistance helps prevent degradation over time, ensuring the integrity of the obturating material and minimizing the risk of microleakage and subsequent failure [18]. Nevertheless, it is crucial to acknowledge that the efficacy of a root canal procedure is contingent upon not only the specific sealer employed but also on a multitude of factors. These factors encompass a thorough biomechanical preparation of the root canal system, the removal of pulp tissue and bacteria completely, and the appropriate filling of the canals [19]. Furthermore, the total strength and FR of a tooth that has undergone endodontic therapy can be influenced by various factors, including the residual dental structure, the extent of the carious lesion or damage, and the kind of restorative materials applied to the tooth subsequent to the endodontic procedure.

Choosing the right gutta-percha and sealer combination can enhance the tooth's fracture resistance. For example, compared to single-cone obturation, investigations have demonstrated that cold lateral compaction and warm vertical compaction can increase fracture resistance [20,21]. The presence of a post in the root-filled tooth can impact its fracture resistance. Some research demonstrated that teeth with intraarticular posts may have lower fracture resistance compared to those without posts [22,23]. However, it's essential to consider the individual case and the type of post used. The overall FR of root-filled teeth is significantly impacted by several factors, including the sheer amount and fracture toughness of the obturated tooth. Teeth with more intact coronal tooth structure tend to have better resistance to fractures. Some studies have explored the utilization of fiber posts and other reinforcement materials to increase the FR of the obturated teeth [24,25]. These reinforcement techniques can provide additional support to the tooth and improve its resistance to fractures. Long-term clinical studies are essential to assess root-filled teeth' durability and fracture resistance over time. However, research in this area is challenging due to the lengthy follow-up periods required. Although there is evidence that AH+ improves the FR of obturated teeth, it is crucial to remain cognizant that the effectiveness of endodontic therapeutic procedures is dependent on an array of factors in addition to the sealer. The success of an endodontic procedure depends on several factors, including thorough biomechanical preparation of the canal, careful extirpation of the infected pulp tissue and bacteria, and precise obturation [18]. Dentists must use proper techniques and high-quality materials and consider the overall condition of the tooth to optimize the chances of a successful and durable outcome.

## Conclusions

Within the limitations of the present study, all groups with sealers evaluated under laboratory conditions successfully strengthened the structure of the teeth. However, AH plus sealer was found to strengthen the tooth structure more effectively than the Resilon-Epiphany system but there was an insignificant difference with the AH 26 sealer. Both sealers are epoxy resin-based, and they are likely to provide relatively similar fracture resistance due to their adhesive properties and ability to form strong bonds with the tooth structure. Eventually, the choice between AH 26 and AH+ may depend on the specific case, the preference of the clinician, and the obturation technique being employed. Both sealers have their strengths and have been widely used in endodontic practice. As with any dental material, dental professionals must follow proper protocols and techniques to ensure the best treatment outcomes. However, further studies are needed to evaluate the success of both AH plus and the Resilon-Epiphany system under clinical conditions.
